# Perceived treatment urgency of common mental disorders in the German population

**DOI:** 10.1038/s41598-023-49969-3

**Published:** 2023-12-19

**Authors:** Sarah Koens, Jens Klein, Martin Scherer, Annette Strauß, Martin Härter, Ingo Schäfer, Daniel Lüdecke, Olaf von dem Knesebeck

**Affiliations:** 1 Institute of Medical Sociology, University Medical Center Hamburg-Eppendorf, Martinistr. 52, 20246 Hamburg, Germany; 2 Department of General Practice and Primary Care, University Medical Center Hamburg-Eppendorf, Martinistr. 52, 20246 Hamburg, Germany; 3 Department of Medical Psychology, University Medical Center Hamburg-Eppendorf, Martinistr. 52, 20246 Hamburg, Germany; 4 Department of Psychiatry and Psychotherapy, University Medical Center Hamburg-Eppendorf, Martinistr. 52, 20246 Hamburg, Germany

**Keywords:** Psychology, Health care

## Abstract

Perceived treatment urgency of mental disorders are important as they determine utilization of health care. The aim was to analyze variations in perceived treatment urgency in cases of psychosis (adolescents), alcoholism (adults), and depression (older adults) with two levels of severity each by characteristics of the case and the respondents. A telephone survey (N = 1200) with vignettes describing cases of psychosis, alcoholism, and depression was conducted in Hamburg, Germany. Vignettes varied by symptom severity and sex. Perceived treatment urgency was assessed by three items. A sum scale was calculated. Linear regression models were computed to analyze differences in perceived urgency by characteristics of the case (severity, sex) and the respondents (sex, age, education, migration background, illness recognition, personal affliction). Perceived treatment urgency was significantly higher in severe cases and varied by education. Additionally, regarding psychosis, estimated urgency varied significantly by correct illness recognition. With regard to depression, perceived urgency differed significantly by age and correct illness recognition. Interaction effects between case severity and sociodemographic characteristics of the respondents, personal affliction, and correct recognition of the disorder were found. The identified differences should be considered in the development of interventions on mental health literacy with regard to adequate urgency assessment.

## Introduction

The concept of mental health literacy [MHL], introduced by Jorm et al.^1^ refers “to knowledge and beliefs about mental disorders which aid their recognition, management or prevention”^1^. MHL includes the ability to recognize specific mental disorders, knowledge and beliefs about causes, risk factors, and effectiveness of self-helping interventions^1–3^. MHL is an evolving concept and also includes knowledge of “when and where to seek help”^4^. Thus, MHL refers to more than a test of knowledge as it also includes aspects of management and the ability to seek adequate help^1,2^. In the context of MHL, perception of urgency should also be considered as a relevant aspect as it can result in insufficient health care utilization and subsequently in increased health risks. In this context, perceptions of urgency include beliefs about whether a specific condition is perceived as an emergency, if immediate treatment is regarded as essential, and whether a hospital visit is perceived as necessary.

MHL can be assessed regarding specific mental disorders. The study is focused on the three different illnesses psychosis, alcoholism, and depression. About one to two percent of the German population are affected by psychosis, which often occurs between the ages of 12 and 29 for the first time^5^. Moreover, psychosis is among the most frequent reasons for visiting emergency departments [ED] in children^6^ and paranoid schizophrenia accounted for 14.2% of psychiatric diagnoses in a German ED^7^. Studies from Germany and the European Union showed a 1-year prevalence of alcohol dependency of 3.4%^8,9^. In terms of emergency care, another study found, that alcohol use disorders accounted for 3.8% of all ED visits^10^, and a German study reported that alcohol intoxication accounted for 20.2% of psychiatric diagnosis in an ED^7^. A German study reported a lifetime prevalence of depression of 11.6%^11^. In older persons, depression is the most frequent mental illness and the suicide rate is highest in older persons^12^. The ED is an important contact point for patients with depression^13^ and suicidality was among the most frequent psychiatric diagnoses in an interdisciplinary ED in Germany^7^.

Regarding psychosis, not being able to assess severity of symptoms was identified as a barrier to use help^14^. There are also studies on alcohol-related health literacy, but mostly with a focus on risks of alcohol consumption^15^. For example, a recent review addressed the topic alcohol-related health literacy, but no studies on assessment of urgency in alcohol dependence were found^15^. There are also studies reporting limited health literacy among patients with alcohol use disorders undergoing addiction treatment^16,17^. In terms of depression literacy, several studies on recognition of depression exist. For example, in a German study using vignettes, many respondents were able to recognize depression^18^. With regard to all three mental disorders, there is a lack on research of perceived treatment urgency in the general population. This lack is mainly due to the fact that research on MHL commonly did not specifically address urgency perceptions and that emergency literacy is a fairly new and emerging field of research.

Many patients attend EDs on their own initiative^19^. Perceived severity determines utilization of medical services^20^ and among others, patients’ perception of urgency is a reason to utilize emergency care or an ambulance^19,21–23^. Moreover a study on emergency literacy showed, that many people have difficulties to evaluate whether a health problem is a medical emergency^24^.

Overcrowding of EDs is an issue in several countries^25^ and can lead to negative outcomes in patients like reduced patient satisfaction, increased mortality, and risks for adverse outcomes as well as increased stress in staff^26–28^. On the other hand, many persons with mental disorders do not receive treatment^29–31^, and treatment delay can also have negative consequences for patients^32^. Consequently, it is important to assess perceptions of urgency as perceived need influences utilization of health care in terms of mental disorders^30,33^, and underlying beliefs determine utilization of health care services. Thus, we aimed to examine perceived treatment urgency in three prevalent mental disorders, psychosis (adolescent), alcoholism (adult), and depression (older adult) with two levels of severity each. More specifically, the following research questions were addressed:

1. How does the public perceive urgency of three different mental illnesses (psychosis, alcoholism, and depression) with differing levels of severity?
2. Does perceived urgency differ according to sex of the afflicted person, symptom severity and sociodemographic characteristics of the respondents?
3. Does the severity of symptoms interact with sex of the afflicted person and characteristics of the respondents regarding perceived urgency?

## Methods

### Study design and sample

Analyses are based on a cross-sectional survey via computer-assisted telephone interviews (CATI) conducted in Hamburg, a large city of about 1.8 million inhabitants in the northern part of Germany, between October and December 2021. A random sample of people aged 18 years and older was used comprising all possible telephone numbers in Hamburg. Non-registered numbers were included via random digital dialling^34^. The sampling frame of this procedure was based on the range of numbers available in the German telephone network. This range of numbers covers all possible telephone numbers in Germany, whether actually in use or not. Repeated calls were made on different weekdays. To choose the target person on the household level, Kish selection grid was applied^35^. Accordingly, all eligible household members were listed and the target person (i.e. the respondent) was randomly selected. Interviews were conducted by trained interviewers.

In the survey, 48 vignettes were used to assess perceived treatment urgency of different medical complaints (please see below). To identify medium-sized differences, a number of n = 50 participants per vignette was considered sufficient (i.e. total N = 2400). This estimation was based on experiences with previous research^36^. In the net sample, 5222 randomly selected persons (telephone numbers) were included. Of these, 1188 (22.8%) could not be reached, and 1630 (31.2%) refused to participate. A total number of 2404 persons participated. Due to different definitions of eligibility in telephone surveys, different response rates can be calculated^37,38^. Thus, response rate in this survey ranges between 10.9 and 46.0% (American Association of Public Opinion Research RR3^38^ 17.3%). To gain a representative sample of the adult population living in Hamburg, the data set was weighted for sex, age, educational level, and region (district in Hamburg). Therefore, the weighted sample matched the adult population of Hamburg regarding these sociodemographic characteristics^39,40^. For half of the participants (N = 1200), 24 vignettes describing three cases of prevalent mental disorders (psychosis, alcoholism, and depression), each with two levels of severity were utilized (for details please see below). For the other half of the sample, vignettes describing gastrointestinal case stories were presented. The present analyses was exclusively based on the subsample with the vignettes of mental disorders. We decided to specifically focus on these vignettes to reduce complexity.

The respondents were called and informed about the telephone interview and asked for their consent to participate. Since only the participants’ telephone numbers were known and not their names and addresses, verbal informed consent was obtained. Consents and refusals were documented by the interviewers. All procedures were in accordance with the ethical standards of the institutional and/or national research committee and with the 1964 Helsinki Declaration and its later amendments or comparable ethical standards. The study was approved by the Local Psychological Ethics Committee at the Center for Psychosocial Medicine, University Medical Center Hamburg (No. LPEK-0200). All methods were performed in accordance with the relevant guidelines and regulations.

### Vignettes

At the beginning of the interviews, vignettes describing persons with one of the three mental disorders were presented as a stimulus (please see additional file 1). These short case stories were developed in cooperation with twelve clinical and scientific experts (primary care physicians, emergency physicians, geriatricians, pediatricians, psychiatrists, psychologists, and researchers) and patients. In a number of meetings, the developed case scenarios were discussed and revised after each meeting until a consensus was achieved among all experts. It was intended to get a short, but realistic symptom description and a clear distinction between two levels of severity. Based on recommendations of these experts, three common mental illnesses were selected for the vignettes for three age groups: psychosis for the adolescent vignette (15 years old), alcoholism for the middle-aged person (49 years old), and depression for the older person (72 years old). The term psychosis rather designates a syndrome than a disorder, but given its widespread use in clinical practice, preference was given to it over more narrow descriptions like schizophrenia or bipolar disorder. Psychosis was indicated by delusion, hallucination (hearing voices), social withdrawal, and thinking disturbance. Higher severity was indicated by hearing voices giving instructions which could lead to self-harm^5^. Psychosis was chosen as it is among the most frequent reasons for visiting ED in adolescents^6^. Alcoholism was described by drinking alcohol daily over the past time, problems at work and with the spouse, and social withdrawal. To indicate higher severity, a case of withdrawal and delirium was described^41^. Depression was indicated by feeling depressed, sleeping disorders, loss of interest/cheerlessness and concentration difficulties. High severity was indicated by suicidal thoughts^12^. Additionally, vignettes were varied according to sex (male; female), and daytime (Tuesday, 8 a.m.; Tuesday, 8 p.m.). As daytime was not expected to be important for the perception of urgency, this variation of vignette characteristic was not taken into account for further analyses. Adequate reactions to the symptoms in the vignettes were discussed with clinical experts. The experts classified the vignettes with higher severity as emergencies where waiting is not recommended. They suggested to call an ambulance or go to an ED in these severe cases. The less severe cases were not classified as emergencies and the clinical experts recommended not to go to an ED and not to call an ambulance. The resulting vignettes were audio-recorded by a clearly speaking trained person to avoid variations in the presentation of the vignettes. The audio files were directly played to the participants via the computer. To each participant one vignette was presented for several reasons. To increase response rate, the length of the interview was limited, and it was also intended to avoid overburdening the participants. Furthermore, using more than one vignette per participant could influence the response behavior from one vignette to the next. The vignettes were randomly assigned to the participants. We used unlabeled vignettes, i.e. the respondents were not informed about the disorder presented.

### Measures

To assess perceived treatment urgency of the symptoms presented in the vignettes, three items were developed based on previous studies among patients using emergency care^19,21^: (1) “The disease can have severe consequences if not treated immediately” (2) “If such complaints are present, it is an emergency” (3) “With such complaints, it is better to go straight to the hospital”. The respondents could indicate their level of agreement or disagreement with these statements on a four-point Likert scale (fully agree, rather agree, rather disagree, fully disagree). With the three items, a principal component analysis was carried out. All three items loaded on one component with an Eigenvalue of 1.87 (explained variance: 62.29%, Cronbach's α: 0.70). Accordingly, a sum score ranging from 3 to 12 was computed. Higher scores indicate stronger agreement with urgency perceptions.

Regarding the respondents, the following sociodemographic characteristics were considered: sex (male/female), age, education [up to 9 years of schooling (low)/10 years of schooling (middle)/at least 12 years of schooling (high)], and migration background (no/1st generation/2nd generation). People were considered to have a migration background if they themselves or at least one parent were not born in Germany. Migrants, who were not born in Germany, were considered as 1st generation migrants, while people, who were born in Germany, with at least one parent not born in Germany, were considered as 2nd generation migrants. Additionally, the respondents were asked, which disorder was presented in the vignettes, to assess whether the illness was recognized correctly (yes/no), and they were asked whether they or their child/children have ever been affected by the complaints, which were described in the vignettes (yes/no).

### Analyses

Analyses were based on the group of participants, to whom vignettes describing mental disorders (psychosis, alcoholism, and depression) were presented (N = 1200). As descriptive results, relative frequencies of agreement (fully agree and rather agree summed up) with the three items and mean values with standard deviation [sd] of the sum score were calculated. To analyse associations of sociodemographic characteristics of the respondents and vignette characteristics with perceived treatment urgency (sum scale), multiple linear regression models were calculated for the three mental illnesses separately. In terms of key assumptions for linear regression, normal distribution of residuals were indicated by histograms and pp-plots while a linear relationship was shown by scatter plots. No auto-correlation was given by very satisfactory results of Durbin-Watson-tests (1.918, 2.188, and 2.041). Furthermore, there was no multicollinearity (VIF: 1.015–2.693), and homoscedasticity was also indicated by scatterplots. Finally, no relevant outliers were found (Cook's distance).Vignette characteristics (sex and severity) and characteristics of the respondents (sex, age, school education, migration background, personal affliction, and recognition of the presented illness) were entered simultaneously into the models. To analyze differences by severity in the vignettes in more detail, interaction analyses were conducted. First, to identify significant two-way interactions, interaction terms with severity of the illness and the other variables in the models were calculated in linear regression models and significant interaction effects are reported. In case of significant interaction terms, estimated marginal means with 95% confidence intervals [CI], pairwise comparison, and Tukey adjusted p values for the respective interactions were computed, to examine the interaction effects in more details.

All analyses were carried out with weighted data and for the three disorders separately (listwise deletion of missing values). Principal component analysis, descriptive analyses, and linear regression models were carried out in the Statistical Package for the Social Sciences (SPSS 27)^42^. Interaction analyses were carried out using the R statistical package^43^ including the package emmeans^44^. p values < 0.05 were considered statistically significant.

## Results

### Sample characteristics

Sample characteristics are shown in Table 1. Mean age of the respondents was 49.45 years; 51.4% were female, and 76.1% had no migration background. Regarding education, 47.0% had 12 years of schooling or more (i.e. high education). In case of the psychosis vignette, 2.1% (high severity) and 2.9% (lower severity) of the respondents or their children had been affected by the symptoms. Regarding the alcoholism vignette, 3.2% (high severity) and 4.8% (lower severity) of the respondents had been affected. With regard to the depression vignette, these rates were 36.7% (high severity) and 37.3% (lower severity). Correct identification of illness ranged between 20.3% (psychosis with lower severity) and 82.6% (depression with high severity).

**Table 1:** Sample characteristics and distribution of variables (weighted data; N = 1200)*.

	n (%) or M ± SD
Sex *(0)*	
Female	617 (51.4)
Male	583 (48.6)
Age (M ± SD) *(0)*	49.5 ± 18.9
Education *(33)*	
Low	313 (26.8)
Middle	305 (26.2)
High	549 (47.0)
Migration background *(19)*	
No	899 (76.1)
1st generation	137 (11.6)
2nd generation	145 (12.3)
Illness recognized *(0)*	
Yes	687 (57.3)
No	513 (42.7)
Personal affliction *(9)*	
Yes	168 (14.1)
No	1022 (85.9)
Urgency of treatment perceptions (range 3–12)^+^ (M ± SD) *(45)*	
Psychosis	
Lower severity	8.24 ± 1.56
Higher severity	9.30 ± 1.72
Alcoholism	
Lower severity	7.81 ± 2.02
Higher severity	8.50 ± 2.11
Depression	
Lower severity	7.44 ± 1.82
Higher severity	8.37 ± 1.87

*Number of missing cases in brackets in italics.

^+^Higher values indicate higher perception of urgency.

### Perception of urgency

**Figure 1:**
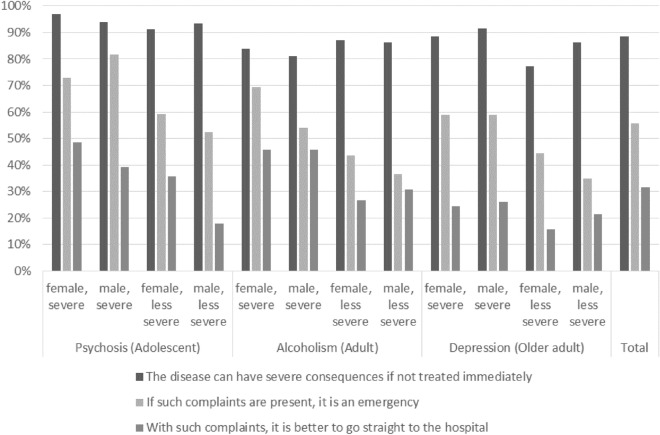
Agreement (fully agree and rather agree summed up) with perceptions of urgency (%), N = 1200 (total); n = 100 (per vignette).

Figure 1 shows the agreement with the three items measuring perceived treatment urgency by severity and sex in the vignettes. For the item “The disease can have severe consequences if not treated immediately”, agreement (percentage of respondents who rather and fully agreed summed up) was comparatively high across all vignettes [ranging from 77.3% (female depression vignette with lower severity) to 97.0% (female severe psychosis vignette)]. Agreement with the item “If such complaints are present, it is an emergency” was lower, with larger differences between the vignettes [range 34.7% (male depression vignette with lower severity) to 81.7% (male severe psychosis vignette)]. Agreement with the item “With such complaints, it is better to go straight to the hospital” was lowest with a range between 15.7% (female depression vignette with lower severity) and 48.5% (female severe psychosis vignette).

Mean score of urgency was 9.30 (sd 1.72) for psychosis with high severity and 8.24 (sd 1.56) for lower severity. In terms of alcoholism, mean score of urgency was 8.50 (sd 2.11) (high severity) and 7.81 (sd 2.02) (lower severity). Regarding depression, mean score of urgency was 8.37 (sd 1.87) for high severity and 7.44 (sd 1.82) for lower severity. The regression analysis showed that respondents rated urgency (sum scale) of the less severe symptoms significantly lower for all three mental disorders (Table 2). In terms of the psychosis vignette, perceived urgency was significantly higher in respondents with high education and in those, who recognized the symptoms correctly. Urgency was perceived slightly higher for female vignettes (not significant). Regarding the alcoholism vignette, estimated urgency was significantly lower in respondents with 10 years of school education compared to respondents with lower education. Perceived urgency was slightly lower for respondents with a 2nd generation migration background and in persons, who recognized the presented illness correctly (both not significant). In terms of the depression vignette, perceived urgency was significantly lower in older respondents and in respondents with middle and high education compared to lower education. Additionally, perceived urgency was significantly lower in persons, who recognized the symptoms correctly.

**Table 2:** Perceived urgency (sum scale) in psychosis, alcoholism, and depression, linear regression.

	Psychosis (adolescent), N = 371			Alcoholism (adult), N = 375			Depression (older adult), N = 356		
	B (95% CI)	β	p	B (95% CI)	β	p	B (95% CI)	β	p
Vignette characteristics									
Sex: female	0.323 (− 0.021 to 0.666)	0.094	0.066	0.356 (− 0.068 to 0.781)	0.085	0.100	− 0.242 (− 0.603 to 0.119)	− 0.063	0.188
Severity: lower severity	− **1.031 (**− **1.376 to **− **0.686)**	− **0.299**	** < 0.001**	− **0.869 (**− **1.295 to **− **0.443)**	− **0.206**	** < 0.001**	− **1.080 (**− **1.446 to **− **0.715)**	− **0.284**	** < 0.001**
Respondents characteristics									
Sex: female	0.002 (− 0.337 to 0.340)	0.000	0.992	− 0.310 (− 0.752 to 0.131)	− 0.074	0.168	− 0.135 (− 0.514 to 0.245)	− 0.035	0.485
Age	0.003 (− 0.006 to 0.013)	0.037	0.491	− 0.002 (− 0.015 to 0.010)	− 0.021	0.712	− **0.031 (**− **0.042 to **− **0.020)**	− **0.311**	** < 0.001**
School education: middle*	0.352 (− 0.143 to 0.848)	0.082	0.163	− **0.784 (**− **1.403 to **− **0.165)**	− **0.165**	**0.013**	− **1.277** **(**− **1.803 to **− **0.751)**	− **0.310**	** < 0.001**
School education: high*	**0.593 (0.186** to **1.000)**	**0.172**	**0.004**	− 0.363 (− 0.924 to 0.199)	− 0.086	0.205	− **1.631 (**− **2.151 to **− **1.112)**	− **0.426**	** < 0.001**
Migration background: 2nd generation^+^	− 0.040 (− 0.582 to 0.503)	− 0.008	0.886	− 0.642 (− 1.301 to 0.018)	− 0.104	0.056	0.553 (− 0.029 to 1.135)	0.094	0.062
Migration background: 1st generation^+^	0.337 (− 0.172 to 0.847)	0.066	0.194	0.185 (− 0.489 to 0.859)	0.030	0.590	− 0.152 (− 0.848 to 0.545)	− 0.021	0.669
Illness recognized: yes	**0.440 (0.049** to **0.831)**	**0.114**	**0.028**	− 0.444 (− 0.951 to 0.063)	− 0.090	0.086	− **0.611 (**− **1.064 to **− **0.159)**	− **0.141**	**0.008**
Personal affliction: yes	− 0.065 (− 1.255 to 1.125)	− 0.005	0.915	0.754 (− 0.324 to 1.833)	0.070	0.170	0.024 (− 0.361 to 0.409)	0.006	0.904
R^2^ adjusted	0.101			0.060			0.209		

Significant values are in [bold].

*Reference: lower education.

^+^Reference: no migration background.

### Interactions

For the psychosis vignette, significant interaction effects between symptom severity and education (middle: p = 0.255; high: p < 0.001) and between severity and personal affliction (p = 0.026) were found. Table 3 shows estimated marginal means from the interaction analysis to examine the significant interactions in more detail. There were significant differences by education for the vignette with high severity but not for the vignette with lower severity. Additionally, in terms of high severity, perceived urgency was slightly higher in respondents who had been affected while perceived urgency was slightly lower in respondents who had been affected in terms of lower severity.

**Table 3:** Estimated marginal means (95% CI) for perceived urgency (sum scale) in psychosis (adolescent), N = 371, range 3–12.

	High severity	Lower severity
School education		
Low	8.87 (8.18–9.56)	8.89 (8.07–9.70)
Middle	9.39 (8.56–10.21)	8.83 (8.03–9.64)
High	10.21 (9.49–10.92)	8.44 (7.74–9.14)
p (low vs. middle)*	0.298	0.988
p (low vs. high)*	< 0.001	0.327
p (middle vs. high)*	0.033	0.407
p (interaction term severity × middle educational level)	0.255	
P (interaction term severity × high educational level)	< 0.001	
Personal affliction		
No	9.38 (9.08–9.68)	8.41 (8.07–8.74)
Yes	10.55 (8.93–12.16)	6.89 (5.15–8.63)
p (no vs. yes)*	0.480	0.319
p (interaction term severity × personal affliction)	0.026	

*Tukey adjusted.

Regarding the alcoholism vignette, significant interactions between severity of the symptoms and education (middle: p = 0.354; high: p = 0.012) and between severity and recognition of the disorder (p = 0.002) were identified. Perceived urgency differed significantly by education in symptoms with lower severity, but not for alcoholism with high severity (Table 4). For symptoms with lower severity, respondents, who recognized the symptoms correctly, rated the urgency lower, while perceived urgency did not differ in terms of symptoms with high severity.

**Table 4:** Estimated marginal means (95% CI) for perceived urgency (sum scale) in alcoholism (adult), N = 375, range 3–12.

	High severity	Lower severity
School education		
Low	8.90 (8.05–9.74)	8.84 (8.07–9.62)
Middle	8.47 (7.65–9.28)	7.85 (7.02–8.68)
High	9.29 (8.55–10.04)	7.89 (7.19–8.59)
p (low vs. middle)*	0.601	0.053
p (low vs. high)*	0.603	0.026
p (middle vs. high)*	0.060	0.994
p (interaction term severity × middle educational level)	0.354	
p (interaction term severity × high educational level)	0.012	
No illness recognized		
No	8.32 (7.40–9.24)	8.70 (7.99–9.42)
Yes	8.86 (8.22–9.49)	7.63 (6.99–8.28)
p (no vs. yes)*	0.556	0.007
p (interaction term severity × illness recognition)	0.002	

*Tukey adjusted.

Regarding the depression vignette, the analysis showed significant interactions between severity in the vignette and sex of the respondents (p = 0.034), severity and personal affliction (p = 0.008), and between severity and migration background (2nd generation: p < 0.001; 1st generation: p = 0.604). More specifically, for the vignette with high severity, perceived urgency differed by migration history, whereas there was no difference in less severe symptoms (Table 5). Additionally, there was a larger difference in perceived treatment urgency between depression with high severity and lower severity in respondents who had been affected by the symptoms themselves compared to respondents who had not been affected by the symptoms themselves.

**Table 5:** Estimated marginal means (95% CI) for perceived urgency (sum scale) in depression (older adult), N = 356, range 3–12.

	High severity	Lower severity
Sex (respondents)		
Male	8.68 (8.20–9.16)	8.08 (7.60–8.56)
Female	8.91 (8.49–9.34)	7.51 (7.10–7.92)
p (male vs. female)*	0.800	0.178
p (interaction term severity × sex)	0.034	
Migration background		
No migration background	8.56 (8.25–8.87)	7.79 (7.49–8.08)
1st generation	8.59 (7.64–9.54)	7.46 (6.56–8.36)
2nd generation	10.24 (9.49–10.98)	7.10 (6.34–7.86)
p (no migration background vs. 1st generation)*	0.998	0.773
p (no migration background vs. 2nd generation)*	< 0.001	0.223
p (1st generation vs. 2nd generation)*	0.021	0.824
p (interaction term severity × 1st generation)	0.604	
p (interaction term severity × 2nd generation)	< 0.001	
Personal affliction		
No	8.63 (8.22–9.03)	7.96 (7.56–8.36)
Yes	9.12 (8.63–9.61)	7.43 (6.94–7.92)
p (no vs. yes)*	0.238	0.245
p (interaction term severity × personal affliction)	0.008	

*Tukey adjusted.

## Discussion

We analyzed perceived treatment urgency in three common mental disorders, psychosis, alcoholism, and depression, with two different levels of severity each. For all three disorders, respondents rated urgency higher when a vignette with high severity was presented. The clinical experts involved in the vignette development classified the symptoms with high severity as emergencies and recommended to utilize the ED or an ambulance while they recommended not to use the ED or an ambulance in the cases with lower severity. Therefore, this is an important result, as it indicates that the majority of respondents were able to distinguish between an emergency with high severity and cases with lower severity. Additionally, we identified variations in perceptions of urgency by education of the respondents and correct recognition of the symptoms in terms of all three mental disorders, variations by age, sex, and migration background in terms of the depression vignette and by personal affliction in terms of the psychosis and the depression vignette.

To our knowledge, this is the first study examining perceived treatment urgency in different mental disorders. Thus, our results are only comparable to previous studies to a limited extend. Our results showed that urgency in the psychosis vignette was estimated higher by respondents with higher school education. Moreover, the interaction analysis showed that in terms of high severity, respondents with higher school education rated the symptoms more urgent compared to respondents with low and middle education. This indicates that a higher education is associated with a more adequate assessment of urgency and a better recognition of an emergency. In terms of the alcoholism vignette, respondents with medium school education rated urgency lower. According to the interaction analysis, respondents with high education estimated urgency lower compared to respondents with low education in a less severe case. Other studies reported a higher MHL in people with higher education^45–47^. This fits our results that indicate a more adequate assessment of urgency in persons with higher education. In terms of the depression vignette, perceived urgency was lower in respondents with medium and higher school education.

According to our results, sex of the affected person was not associated with perceptions of urgency in cases of psychosis, alcoholism, and depression. Regarding sex of the respondents, there was only one significant interaction with severity of the symptoms indicating a more adequate perception of urgency in women compared to men in the case of depression. With regard to psychosis and alcoholism, sex of the respondents was not associated with urgency perceptions. There are other studies indicating a higher MHL in women^46,48–51^, but other results showed no sex differences in MHL^52^. One vignette study reported a higher depression literacy regarding recognition but no sex differences with regard to knowledge on treatment^45^. Regarding the depression vignette, urgency was rated lower with increasing age of the respondents. In this regard, other studies reported inconsistent results on MHL^45–47,53,54^. One significant interaction between severity of the symptoms and migration background indicates that respondents with a 2nd generation migration background perceived urgency higher in terms of a depression with high severity, while there were no significant differences in a less severe case. Otherwise, migration background was not associated with perceived treatment urgency.

Urgency in the psychosis vignette was estimated higher by respondents who identified the symptoms correctly. Regarding depression, urgency was rated lower by respondents, who recognized the symptoms correctly. With regard to the alcoholism vignette, the interaction analysis showed that respondents who recognized the symptoms correctly rated the urgency of the mental disorder more often adequately. This indicates that correct identification of symptoms might be associated with adequate assessment of urgency. Correct recognition of mental disorders is a component of MHL^1–3^. In terms of the psychosis and the depression vignettes, the results of the interaction analyses indicate, that respondents, who were affected by the symptoms in the past, rated urgency of the disorders more often adequately. This is an important result, as it indicates that familiarity with symptoms might be associated with adequate estimation of urgency in mental illnesses. A previous study reported a higher depression literacy in people who were affected by a depression and had treatment but not in people who had depression but no treatment^47^. Other studies also reported a higher MHL in afflicted persons or people in proximity to afflicted persons^52,55^. However, according to one of these studies this was only true for depression and not for schizophrenia^52^.

With regard to our findings, some methodological aspects have to be considered. First, a response rate between 10.9 and 46% (depending on definition of eligibility) is considered acceptable compared to other telephone surveys^56^, but a selection bias cannot be ruled out, as more than half of the randomly selected people refused to participate or were not available and there may be differences between participants and non-responders regarding their perceived treatment urgency of mental disorders. Another limitation concerning the recruitment is that we only used landline numbers and therefore people without landline numbers could not participate in the survey. This was necessary as we used a regional sample and mobile numbers in Germany cannot be assigned to regions. There was no information available about how many people in the region still use landline numbers which potentially limits quality of the sample. However, comparison of our weighted data with official statistics regarding sex, age, and school education revealed no significant differences^39,40^. Furthermore, vignettes are often used to measure public beliefs and attitudes in public mental health research. However, the vignettes had to be short, because they had to be remembered through the first part of the telephone interview. The vignettes were developed in cooperation with clinical experts to describe the cases realistically. Nevertheless, it has to be considered that participants may perceive urgency differently if they were really affected by the disorders in question. Moreover, regarding measurement of perceptions, we decided to use a four-point Likert scale without a mid-category as it could discourage from taking sides resulting in forgone data, and attraction to mid-category differs between certain subgroups^57^. On the other hand, a category “don’t know” was offered (treated as missing value in the analyses). For the three items used, number of respondents who chose this category was rather low and ranged between n = 19 and n = 33. Nevertheless, we are aware that ambivalence could not be sufficiently indicated. As agreed upon with clinical experts, three different disorders that have high prevalence and are typical for the respective age groups and that are relevant regarding emergency care were chosen for the vignettes. Although care was taken to ensure that the illnesses in the vignettes had a comparable urgency for treatment, the results are not comparable for the three age groups and we cannot tell, whether differences between the disorders may also be due to the different age of the described person. As there were no validated instruments to assess public perceptions of urgency, we developed three items and carried out a principal component analysis. Internal consistency of the scale was acceptable (Cronbach’s α = 0.70)^58^. Finally, the study was only conducted in one big city (1.8 million inhabitants) in Northern Germany (Hamburg). Even though, this fact may not be crucial for urgency of treatment perceptions, our results cannot be generalized to Germany, other countries and regions or smaller cities.

## Conclusions

In conclusion, our study indicates that it is possible for the general population to differentiate between two levels of urgency in cases of three common mental disorders. Moreover, we identified differences in perceived treatment urgency especially by school education, correct illness recognition, and personal affliction. Our results on variations in perceptions of urgency are important as perceptions of need are associated with help seeking preferences^30,33^ and self-perceived urgency is associated with ED and ambulance utilization^19,21,23^. In light of frequent utilization of EDs on the one hand and treatment delay on the other hand^59^, adequate estimation of urgency might be relevant for adequate help seeking behavior in cases of mental disorders and for the planning of health care for people affected by mental disorders. The identified differences should be taken into account in the planning of interventions to promote MHL with regard to assessment of urgency of mental disorders, to prevent inadequate utilization of health care services.

### Supplementary Information

Supplementary Information.

## Data Availability

Data are available from the corresponding author on reasonable request.
