# Supplementary material for: Perceived treatment urgency of common mental disorders in the German population
Source: Sci Rep. 2023 Dec 19;13:22711. doi: 10.1038/s41598-023-49969-3 (PMC10733386; doi:10.1038/s41598-023-49969-3)
Supplement: Supplementary file 1 — Supplementary Information. [file 41598_2023_49969_MOESM1_ESM.pdf]

Perceived treatment urgency of common mental disorders in the German population

Sarah Koens[1], Jens Klein[1*], Martin Scherer[2], Annette Strauß[2], Martin Härter[3], Ingo Schäfer[4], Daniel Lüdecke[1], Olaf von dem Knesebeck[1]

[1] Institute of Medical Sociology, University Medical Center Hamburg-Eppendorf, Martinistr. 52, 20246 Hamburg, Germany

[2] Department of General Practice and Primary Care, University Medical Center Hamburg-Eppendorf, Martinistr. 52, 20246 Hamburg, Germany

[3] Department of Medical Psychology, University Medical Center Hamburg-Eppendorf, Martinistr. 52, 20246 Hamburg, Germany

[4] Department of Psychiatry and Psychotherapy, University Medical Center Hamburg-Eppendorf, Martinistr. 52, 20246 Hamburg, Germany

*Corresponding author

Case vignettes

Psychosis (adolescent, 15 years)
**High severity**<br>*It is Tuesday morning, 8 a.m./ It is Tuesday evening, 8 p.m. Paula S./ Lukas P., 15 years old, pupil,*<br>has been withdrawing more and more into herself for half a year now, avoids everyone and has the impression that others can read her mind. She is often agitated and absent-minded. For some time now, Paula has felt threatened and has the feeling that she is being followed. She also hears voices that disturb her thinking and give her instructions. Today the voices ordered her to jump out of the window. She was able to resist, but fears that she will not be able to do so for long. Her parents are very worried.
**Lower severity**<br>*It is Tuesday morning, 8 a.m./ It is Tuesday evening, 8 p.m. Paula S./ Lukas P., 15 years old, pupil,*<br>has been withdrawing more and more into herself for half a year now, avoids everyone and has the impression that others can read her mind. She is often agitated and absent-minded. For some time now, Paula has felt threatened and has the feeling that she is being followed. She also hears voices that disturb her thinking and comment on her actions. Her parents are worried.

**Alcoholism (adult, 49 years)**
**High severity**<br>*It is Tuesday morning/Tuesday evening, 8 a.m. Melanie P./ Stefan D., 49 years, administrative employee,*<br>has increasingly been drinking more alcohol over the past months, lately about 2 bottles of wine every day, frequently also some hard liquor in the evenings. Mrs. P already drinks a glass of vodka in the mornings to avoid going to work shaky. After a written warning at her workplace and a severe dispute with her husband two days ago, she decided to get to grips with her life and since then has not drunk alcohol anymore. Today Mrs. P. cannot go to work, for hours she has been fidgety, jumpy and she perspires, even though it is chilly. Her husband is very worried, also because Mrs. P. imagines to see mice scuttling around that do not exist.
**Lower severity**<br>*It is Tuesday morning, 8 a.m./ It is Tuesday evening, 8 p.m. Melanie P./ Stefan D., 49 years, administrative employee,*<br>has, in recent months, been drinking increasingly more alcohol than before, lately two bottles of wine every day. Mrs. P already drinks a glass of vodka in the mornings to avoid going to work shaky. She has retired from the sports club in which she used to be very active. She does not want to meet anybody. At work there already was a written warning. Her husband is worried, because he cannot talk properly with her anymore. There are a lot of arguments.

Depression (older adult, 72 years)
**High severity**<br><br>*It is Tuesday morning, 8 a.m./It is Tuesday evening, 8 p.m. Hildegard S./ Helmut K., 72 year, pensioner,*<br><br>has been feeling so down all the time for several months now that nothing can cheer her up. Ms. S. no longer has anything that she enjoys or is happy about. It takes her a long time to fall asleep in the evenings and she wakes up frequently at night. In the mornings, she is always tired and weak, and cannot concentrate at all. She feels she is not good enough and doubts that her life still has any meaning. Her daughter, who rarely visits, is very worried because Ms. S. has told her that she is currently thinking about taking her life with sleeping pills.
**Lower severity**<br><br>*It is Tuesday morning, 8 a.m./It is Tuesday evening, 8 p.m. Hildegard S./ Helmut K., 72 year, pensioner,*<br><br>has often been feeling so down for several months now that nothing can cheer her up. Ms. S. has lost interest in everyday things. In the evenings, she often has difficulties falling asleep and feels tired and weak in the mornings, and cannot concentrate. She feels she is not good enough. All in all, Ms. S. has great problems managing her day-to-day life. Her daughter, who rarely visits, is worried to see Ms. S. like this.
